# Biography: George A. Fielding, MBBS FRACS FRCS (Eng) FRCS (Glas Hon)

**DOI:** 10.1007/s11695-018-3315-8

**Published:** 2018-06-04

**Authors:** George A. Fielding

**Affiliations:** Department of Surgery, NYU, 530 First Ave., Suite 10S, New York, NY 10016 USA


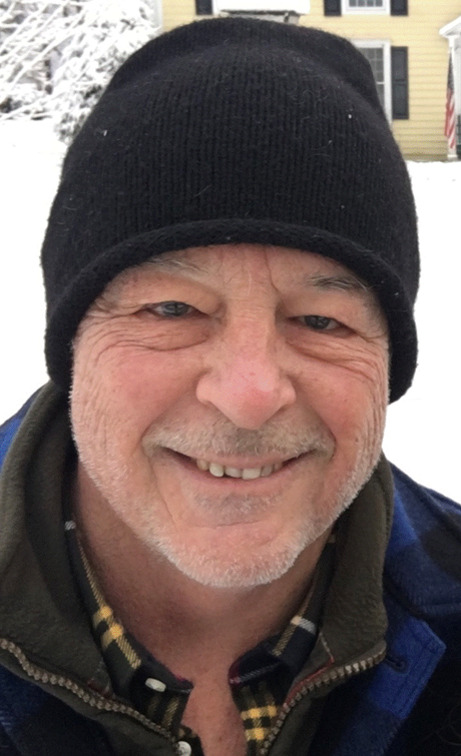
Dr. George Fielding is a general and bariatric surgeon, and the Ira and Nikki Harris Professor of Surgery at NYU Langone Medical Center in New York.

Beginning in 1980, while still in his surgical residency at the Royal Brisbane Hospital in Australia, Professor Fielding developed interests in both hepatobiliary and pancreatic surgery. From 1987 to 1989, he performed fellowship training in Glasgow, Gloucester, and Bern. He then returned to Brisbane in 1990 as an attending surgeon.

Professor Fielding was involved in the early days of the development of laparoscopic surgery. He became a noted expert in this young field and went on to teach laparoscopic techniques to surgeons around the world. Professor Fielding has also been very academically productive. He has published more than 150 journal articles, abstracts, and book chapters. He also has spoken at countless international conferences.

In the mid-1990s, Professor Fielding became increasingly interested in bariatric surgery, and he quickly became a world expert on the Laparoscopic Adjustable Gastric Band Lap Band. Professor Fielding was responsible for training thousands of surgeons (including the Editor-In-Chief of this journal) on the proper operative technique for placing the band, and how to properly manage these patients afterwards.

In addition to general bariatric surgery, Professor Fielding has a special interest in pediatric bariatric surgery and revisional bariatric surgery, as well as hiatal hernia surgery and paraesophageal hernia surgery. Over the course of his lengthy, distinguished career, Professor Fielding has performed more than 10,000 bariatric surgeries. The main goal of his life’s work has been caring for people with severe obesity and performing bariatric surgery with skill and compassion.

In 1999, after years of struggling with weight-related problems, Professor Fielding had bariatric surgery himself. This experience gave him a uniquely personal perspective of our field and what trials and challenges patients experience when they decide to undergo surgery. Professor Fielding has continued to do well with his Lap Band, now more than 18 years later.

In 2006, Professor Fielding had a major life change. He moved from his long-time home in Australia to live and work in New York City. This move enabled him to continue enhancing his academic career and to work alongside his soul mate, Dr. Christine Ren.

George Fielding has four children with his first wife Jennifer Duncombe, a doctor in Brisbane, and three grandchildren. He is now married to Christine, with whom he works at NYU. Christine is the division chief, and his boss, but he takes it all in stride. His passions, apart from his family, include playing guitar and piano, drinking Burgundy and bourbon with friends, fly fishing, and duck hunting. He also loves spending time in the great outdoors of Montana where he and Christine own a home.

